# A NEW FOCUS ON FIBER

**DOI:** 10.1016/j.psj.2026.106863

**Published:** 2026-03-27

**Authors:** Caitlin Evans, Amy Petry, Tim Johnson, Michael H. Kogut, Carrie L. Walk, Tara York

**Affiliations:** a AB Vista, Marlborough, Wiltshire, SN8 4AN, UK; b University of Missouri, Columbia, 65211, USA; c College of Veterinary Medicine, University of Minnesota, St. Paul, 55108, USA; d Agricultural Research Center, College Station, TX 77845, USA

**Keywords:** biomarkers, dietary fiber, microbiome, pigs, poultry

## Abstract

Dietary fiber (DF) in poultry nutrition was once viewed mainly as an anti-nutritional factor that reduced energy density and nutrient digestibility. Current research shows DF can play important functional roles in gut health, nutrient utilization, and overall performance when its type and inclusion level are carefully managed. Fiber fractions differ widely in chemical composition and physical properties such as solubility, water-holding capacity, and water-binding capacity. These factors influence digesta viscosity, passage rate, microbial fermentation, and satiety, making precise fiber characterization critical for effective diet formulation. Traditional methods like crude fiber analysis underestimate total fiber and miss key soluble fractions. Modern approaches, including total dietary fiber analysis and near-infrared spectroscopy allow for better quantification of both insoluble and soluble components and support incorporation into real-time feed formulation. By understanding and targeting specific non-starch polysaccharides or oligosaccharides, nutritionists can promote beneficial fermentation, encourage short-chain fatty acid (SCFA) production, and minimize undesirable protein fermentation or pathogen growth. Dietary fiber also supports intestinal barrier function by stimulating gut development, increasing villus height, and fueling epithelial cells through SCFAs. However, poorly balanced fiber can impair nutrient absorption, increase maintenance energy needs, or elevate digesta viscosity, particularly in young birds. Monitoring gut health is now possible by using biomarkers as tools to evaluate intestinal integrity, inflammation, and microbial balance as we investigate the impact of dietary fiber, but further work is needed to standardize these measures and account for flock variability When properly applied, DF can enhance feed efficiency, improve welfare by reducing hunger-driven behaviors, support reproductive performance, and strengthen disease resilience. A data-driven, precision approach combining accurate fiber analysis, enzyme supplementation, microbiome profiling, and non-invasive gut health biomarkers offers the greatest potential to optimize both productivity and sustainability in poultry systems.

The purpose of this symposium was to stimulate discussion and enhance understanding of dietary fiber and its potential benefits in improving poultry production. This paper provides an overview of each contributing author’s role in the symposium. Dr. Caitlin Evans addressed the challenges associated with current fiber nomenclature and emphasized the need to improve analytical methods and overall understanding of fiber. She brings strong credibility to this topic as a Technical Manager for Near-Infrared Spectroscopy and Feed Milling Engineer at AB Vista, and as a Ph.D. graduate of Kansas State University.

Dr. Amy Petry shared her expertise on the impact of fiber in swine nutrition and how these findings can be applied to poultry. She is an Assistant Professor in the Division of Animal Sciences at the University of Missouri, where her research focuses on improving fiber utilization and its effects on energy efficiency and animal health. Dr. Tim Johnson, Professor in the Department of Veterinary and Biomedical Sciences at the University of Minnesota, highlighted the importance of understanding the poultry microbiome and its role in developing strategies to enhance bird performance. Dr. Mike Kogut contributed his expertise as a Research Microbiologist and Lead Scientist with the Food and Feed Safety Research Unit at the Southern Plains Agricultural Research Center.

Finally, Dr. Carrie Walk, Head of Research at AB Vista, along with Dr. Tara York, AB Vista’s Technical Director for North America, integrated the key themes of the symposium, connecting the presented research and practical applications.

## Section 1: Maximizing the benefits of fiber in poultry nutrition: insights and ingredient evaluation

### Caitlin Evans

Fiber plays a complex role in poultry nutrition, and ongoing research continues to refine strategies for its optimal utilization. Once considered an anti-nutritional factor due to its limited digestibility and potential to hinder nutrient absorption, fiber is now recognized for its functional benefits, including improved intestinal integrity, enhanced gut health, and greater digestive efficiency (Jha et al., 2019). To fully harness these benefits, it is essential to accurately characterize fiber composition in feed ingredients.

Poultry diets predominantly consist of cereal grains and plant-based ingredients, each contributing diverse fiber fractions primarily composed of cellulose, hemicellulose, pectin, and lignin. As the potential benefits of specific fiber fractions for the bird become better understood, accurately distinguishing and quantifying these components has grown increasingly important. However, fiber quantification remains analytically challenging due to its chemical complexity. Indeed, fiber is defined by the analytical method applied, making it required to use qualifiers such as “crude” or “neutral detergent” when reporting results (Fahey et al., 2019). To address these limitations, a variety of analytical approaches have been developed, each capturing different fractions of fiber.

The earliest and arguably the most widely utilized method for fiber determination is crude fiber (**CF**). In this method, the sample is solubilized in sequential acid and alkali extractions. CF quantifies primarily insoluble fractions, such as cellulose and lignin, but fails to capture hemicellulose and soluble fiber. As a result, CF values are known to substantially underestimate total dietary fiber (Fahey et al., 2019). Crude fiber, however, remains the legal measure for grains and feed, but provides limited insight into the physiological effects of fiber in poultry diets. To improve on these limitations, detergent system analyses were developed, including neutral detergent fiber (**NDF**) and acid detergent fiber (**ADF**). NDF represents the plant cell wall fraction and includes cellulose, hemicellulose, and lignin, while ADF quantifies cellulose and lignin but excludes hemicellulose. While NDF and ADF offer insights into the structural, insoluble dietary fiber of the ingredient, these methods fail to capture the soluble fiber components most likely to impact digestion in poultry. Today, individual monomeric sugar composition, including both soluble and insoluble fractions, can be quantified using wet chemistry methods and, more recently, near-infrared (NIR). Incorporating these components enables a more comprehensive assessment of fiber, reported as total dietary fiber (TDF). Total dietary fiber (**TDF**) provides a more complete measurement of fiber content, as it encompasses insoluble and soluble non-starch polysaccharides as well as lignin. This broader classification offers greater insight into fiber’s effects on gut health, fermentation, and nutrient absorption. However, TDF methodologies continue to evolve, and caution is needed when interpreting results to account for differences in analytical procedures. As fiber analysis becomes more complex, so do the associated costs and time requirements. To mitigate these challenges, near-infrared spectroscopy (**NIRS**) may offer a rapid and cost-effective alternative to traditional wet chemistry methods for fiber characterization.

As our understanding of fiber’s role in poultry nutrition advances, so must our ability to quantify and evaluate fiber fractions. Achieving the right balance of fiber types is key to optimizing feed formulations across different poultry species and production stages. A deeper understanding of fiber’s influence on nutrient digestibility, energy utilization, and gut health is critical for developing cost-effective feeding programs. A well-managed fiber strategy can enhance feed efficiency and promote gut health to support long-term productivity in poultry production.

## Section 2: From pigs to poultry: The evolution of dietary fiber in monogastric nutrition

### Amy Petry

Dietary fiber has long been considered to have both negative and positive effects in the nutrition of monogastric animals. Historically, it has been regarded primarily as an antinutritional factor that dilutes dietary energy, reduces nutrient digestibility, and limits growth performance. This has resulted in limited inclusions of high-fiber co-products in commercial formulations or acceptance of reduced performance when periods of elevated feed cost dictate their utilization. However, recent research across species demonstrates that DF is far more than inert bulk and an antinutrient. It is a complex dietary component with unique chemical, physical, and physiological properties that, depending on type and inclusion level, can support gastrointestinal health, influence nutrient utilization, modify animal behavior, and enhance long-term productivity of breeding animals. Thus, both perspectives of fiber, as an antinutrient or a functional component, are valid, and the distinction depends on the context in which DF is utilized. The full dimensionality of DF must be considered, and knowledge gained in swine may provide a useful framework for translation to poultry systems. This is particularly relevant for certain disease challenge conditions and broiler breeders and layers, which face challenges related to satiety, fertility, and welfare that closely parallel those observed in gestating sows.

### Characterizing fiber beyond typical analysis

In monogastric nutrition, DF is defined by Codex Alimentarius as “carbohydrate polymers with ten or more monomeric units that are not hydrolyzed by endogenous enzymes in the small intestine,” encompassing both naturally occurring and synthetic polymers (Jones et al., 2014). Fiber is commonly classified in feed ingredients based on solubility and structure, using analytical systems described earlier in these proceedings by Dr. Evans. Physicochemical properties are defined as “the measurable or observable attributes of a substance that pertain to its physical and chemical properties” (Millets, 2019). In the context of DF, these properties include swelling capacity**,** solubility, water-holding capacity (**WHC**), and water-binding capacity (**WBC**), each of which influences digesta viscosity, passage rate, nutrient absorption, and fermentation (Lindberg, 2014). In combination with fiber analysis, evaluating these attributes provides an additional perspective on how fiber functions within the gastrointestinal tract (Patience and Petry, 2019).

Fiber sources with high WHC and WBC retain more water, improve microbial fermentation, increase microbial enzyme interaction, and enhance satiety. These properties provide valuable tools for selecting fiber sources that achieve specific physiological outcomes in monogastric animals. Fiber composition and physicochemical properties vary widely among ingredients (Table 1). Soy hulls (47% TDF) and sugar beet pulp (52% TDF) contain moderate soluble fractions and favorable hydration properties that support fermentation and fecal hydration. Dried distillers grains with solubles (**DDGS**; 34% TDF) and wheat middlings (26% TDF) are dominated by insoluble fiber with low hydration capacity, contributing mainly to bulk. Pea hulls (80% TDF) and lignocellulose (55% TDF) provide the greatest fiber content, with lignocellulose exhibiting particularly high WHC and water swelling capacity (WSC). These contrasts highlight the need to characterize fiber beyond CF values to design diets that have physiological and functional roles in poultry.

**Table 1:** Fiber composition and physicochemical properties of common fiber sources, as-is basisa.

Fiber Source	TDF^b^, %	IDF^c^, %	SDF^d^, %	WHC^e^, ml/g	WBC^f^, ml/g	WSC^g^, ml/g
Rice bran	25	20	5	2	3	3
Wheat midds	26	23	3	4	3	3
DDGS	34	33	1	2	2	3
Soy hulls	47	38	9	7	5	7
Sugar beet pulp	52	44	8	5	4	8
Lignocellulose	55	53	2	12	2	11
Pea hulls	80	72	8	7	4	6

a Represents average of 5 samples per assay conducted at the MU Monogastric Nutrition Lab.

b Total dietary fiber.

c Insoluble dietary fiber.

d Soluble dietary fiber.

e Water holding capacity.

f Water binding capacity.

g Water swelling capacity.

### Physiological and functional roles of dietary fiber

Dietary fiber influences animal health through both fermentative and structural mechanisms, but its effects depend on fiber type, inclusion level, and the specific pathogen challenge. Responses are pathogen-dependent, and there is no single DF solution across contexts. Fermentable DFs are metabolized by the gut microbiota to produce short chain fatty acids (**SCFA**) such as acetate, propionate, and butyrate, which lower luminal pH, strengthen epithelial barriers, and provide energy substrates for intestinal and peripheral tissues (Lindberg, 2014; Molist et al., 2014). In pigs, sources such as sugar beet pulp and resistant starch reduce enterotoxigenic *E. coli* and *Brachyspira hyodysenteriae* infections, improving growth and survival (Li et al., 2020; Helm et al., 2021), while in poultry, alfalfa meal fermentation decreases *Salmonella* colonization in chicks (Escarcha et al., 2012). In contrast, unfermentable DF acts through physical effects on the gastrointestinal tract; coarse wheat bran improves gut development, accelerates intestinal maturation, and reduces pathogen adhesion (Molist et al., 2011; Molist et al., 2014). In both pigs and poultry, these fibers add bulk, stimulate peristalsis, and improve fecal consistency.

Together, fermentable and unfermentable fibers provide complementary benefits, supporting microbial metabolism and immune defense while enhancing gut structure, motility, and pathogen exclusion, but effective application requires tailoring fiber-type to the pathogen challenge and production context. However, the balance between fiber-type is critical for maintaining gut health and nutrient efficiency. Excess soluble DF can elevate digesta viscosity and reduce nutrient digestibility and feed conversion in broilers (Tejeda and Kim, 2021), while insufficient insoluble DF limits gizzard development and compromises digesta flow. Fiber also alters energy requirements and organ development, with inclusion increasing organ weights and maintenance needs, outcomes that can be detrimental in market birds but advantageous in reproductive animals. Collectively, DF can affect both nutrient utilization or support gastrointestinal health and productivity, but its application must be carefully tailored to fiber source, composition, and production context.

### Translational lessons from sows to broiler breeders

Dietary fiber not only affects digestion and health but also has important implications for animal behavior and welfare. In sows, inclusion of high-fiber diets increases satiety, reduces stereotypic behaviors, and lowers mortality associated with lameness in group-housed systems (Jensen et al., 2012; Oelke et al., 2018). These responses are mediated through delayed gastric emptying, stimulation of satiety-related hormones, and fermentation-driven production of metabolites such as acetate and butyrate that enhance fullness (da Silva, 2013). The same mechanisms are highly relevant to poultry systems where feed restriction is routinely practiced. In broiler breeders, DF can reduce hunger-driven behaviors, aggression, and feather pecking. Studies with oat hulls, beet pulp, potato pulp, and wheat bran have shown reductions in aggressive feeding and drinking behaviors, highlighting the role of DF in mitigating welfare concerns (Nielsen et al., 2011).

Lessons from swine nutrition provide a valuable framework for advancing DF strategies in poultry breeders. European data indicate that sow diets include 35 to 50% more fiber than U.S. diets, with demonstrated benefits for satiety, longevity, and reproductive outcomes. Fiber-rich gilt diets improve retention through parity four and reduce sow mortality during gestation (Rosero et al., unpublished; Levia et al., 2025). During the periparturient period, DF alleviates constipation, shortens farrowing duration, and lowers stillborn rates (Feyera et al., 2017). Poultry breeders and layers face parallel challenges related to satiety, fertility, and longevity, and targeted fiber strategies may offer similar benefits. For example, diets with fiber sources high in WHC regulate feed intake and body weight in broiler breeders (Nascimento et al., 2021), while other work shows DF can alter lipid metabolism and modify egg fatty acid composition (Hu et al., 2024). These parallels reinforce that carefully designed fiber strategies may be a strategy to improve both welfare and reproductive performance in poultry systems.

Dietary fiber is not inert bulk but a functional dietary component with roles that extend beyond energy dilution. The effects of DF depend on type, source, inclusion level, and stage of production. In pigs, DF strategies have improved satiety, reduced mortality, enhanced reproductive performance, and supported sow longevity. In poultry, similar approaches have demonstrated benefits for gut health, welfare, and reproductive outcomes, though responses remain variable. Not all fiber is created equal, and current analytical methods are not able to capture the full variability in the fiber composition. To optimize DF utilization in poultry, nutritionists must move beyond crude measures and consider physicochemical properties that determine function. A cross-species approach offers a powerful framework for refining fiber strategies to balance productivity, animal welfare, and sustainability.

## Session 3: Fiber and the microbiome: what’s all the hype?

### Tim Johnson

Efficient food animal production relies on the overall maintenance of health in the animal and promotion of a balanced and efficient gastrointestinal tract. This can be quite complicated because balance in the gut depends on a healthy microflora balance. There are more than 1000 bacterial species in the guts of food production animals, and these species are naturally balanced when animals are raised in low-stress environments with optimal nutrition and environmental conditions. However, in modern production the establishment of a healthy microbiome is often impeded by confinement and lack of beneficial microbes typically present in nature during development. To address these challenges, feed additives are often used to promote the establishment of beneficial microbes.

Dietary fibers used in food animal production include a diverse range of different combinations of monosaccharides to form oligosaccharides, intended to target specific microbes capable of breaking down sugars. The basic concept of using fibers to modulate the gut microbiome is that fibers can be developed to promote the expansion of specific beneficial groups of bacteria. This, in turn, promotes competitive exclusion of potential pathogens in the gut. At the same time, these beneficial bacteria positively stimulate the immune system and produce byproducts such as SCFAs which are used by the host and improve energy utilization. There is evidence that these interactions could influence the gut-brain axis through microbiota-depedent mechanisms involving microbial metabolites and neural signaling pathways (Dalile et al., 2019).

It is generally believed that dietary fiber and other prebiotics have positive effects on the establishment and activity of important beneficial bacteria in the gut, such as *Lactobacillus* and *Bifidobacteria* species, by serving as fermentable substrates that support their growth and function (Gibson et al., 2017). There is evidence to support this theory, but there is less evidence regarding ancillary effects of DF on the gut microbiome. It is also challenging to differentiate between bacteria which are truly commensal and provide some benefit, versus those that are opportunistic and have potential to exacerbate disease problems. For example, *Escherichia coli* includes many strains that range from harmless to pathogenic to the host under different conditions. There is little to no evidence at the bacterial strain level regarding the specificity of DF, and in general, DF should not be specific to certain strains within the same species. Overall, more high-resolution microbiome studies, including functional analysis, are needed. In addition, standardized platforms capable of evaluating multiple products and their combinations are needed. Although dietary fibers show substantial potential for modulating the gut microbiome, limitations in the current peer-reviewed publication landscape restrict comprehensive data sharing and slow the effective dissemination of knowledge in this field.

## Session 4: Intestinal integrity and key biomarkers for performance

### Michael H. Kogut

#### Poultry homeostasis of the intestinal barrier function (gut health)

Gut homeostasis in poultry is a highly regulated balance between the host and the microbiota in which digestion and absorption, epithelial-barrier integrity, and immune surveillance act in concert to maximize nutrient utilization while restraining microbial overgrowth (Table 2) (Kogut and Arsenault, 2016; Kogut, 2025). The intestinal barrier functions as a boundary between the mucosal immune system in the lamina propria and the external environment of the intestinal lumen, which contains a varied array of microorganisms and ingested environmental factors, including pathogens, feed components, toxins, and other foreign substances (Barekartain et al., 2021). The intestinal barrier has a leading role in regulating the controlled interaction between luminal factors and the intestinal immune system. The intestinal barrier system is composed of a number of different obstruction components, including (1) a microbiological barrier that consists of the commensal microbes which separates the luminal contents from intestine, (2) a chemical barrier which consists of the adhesive mucous gel layer that contains mucins, antimicrobial peptides and mucosal IgA, (3) the physical barrier which consists of a single cell layer of specialized epithelial cells including *enterocytes* with digestive (nutrient/H_2_O absorption), metabolic (maintenance of luminal oxygen levels) and immune (innate immune pattern recognition receptors, cytokine production) functions, *Paneth cells* (production of antimicrobial peptides), *goblet cells* (mucin secretion), *enteroendocrine cells* (secrete hormones regulating gut physiology) and *Tuft cells* (chemosensory activity). The cells of the epithelial layer are bound together and sealed via series of proteins called tight junctions. These proteins are crucial for maintaining the gut barrier function by sealing the intracellular spaces between cells, regulating the passage of ions, water, and molecules through transcellular pathways (Awad et al., 2017; Suzuki, 2020; Cuccato et al., 2022) and preventing the entry of pathogens and toxins (Emami et al., 2019; Cuccato et al., 2022). The fourth barrier (4) in the lamina propria beneath the epithelia cell layer is the immunological barrier holding the humoral and cellular components of the mucosal immune system (Kogut et al., 2020; Wickramasuriya et al., 2022). The function of the mucosal immune system is twofold: defenses against pathogens and induce and keep a state of tolerance to the resident commensal microbes (Tian et al., 2024).

**Table 2:** Homeostasis of the intestinal barrier function.

Intestinal compartment	Host function	Immune characteristics	Microbiota function
Duodenum	Feed digestion: pancreatic enzymes & bile Protein digestion (60%): protein absorption (7%)	Decreasing gradient down the intestine: High oxygen levels Th17 T cells High number of IELs High luminal IgA High AMP production Thin mucus layer	Degradation of plant polysaccharides Lipid metabolism Amino acid metabolism Production of secondary bile salts
Jejunum	Absorption of amino acids, di-& tripeptides, most fatty acids, sugars Protein digestion (25%): protein absorption (63%)		
Ileum	Absorption of linoleic, stearic, palmitic acids Protein digestion (8%): protein absorption (13%)		
Cecum	Fermentation of NSP^a^, cellulose, resistant starches Nitrogen recycling Detoxification (oxidoreductases) Vitamin production Absorption of sugars and some amino acids Water and electrolyte reabsorption from digesta	Thicker mucus layer = increase in goblet cells Low oxygen levels (anaerobic) Secondary bile acids High small chain fatty acids (SCFA^b^) T regulatory cells Increase in B cells = IgA plasma cells	SCFA production Hydrogen consumption Polysaccharide utilization Vitamin synthesis (riboflavin, panthothenic acid, nicotinic acid Energy metabolism Secondary metabolite synthesis

a Non-starch polysaccharides.

b Short chain fatty acids.

#### Loss of intestinal barrier integrity and leaky gut

Loss of intestinal barrier integrity leads to increased intestinal permeability where the non-mediated diffusion of large, normally restricted molecules, including allowing toxins and infectious agents, to “leak” from the intestinal lumen to the circulatory system (Lambert, 2009). This condition is commonly referred to as “leaky gut” (Quigley, 2017; Mu et al., 2017; Chelakkot et al., 2018). In poultry, "leaky gut" refers to a condition where the intestinal barrier is compromised, allowing unwanted substances like bacteria and toxins to pass into the bloodstream. Leaky gut is linked to a disruption of tight junction proteins, which normally function as a "glue" to seal the intestinal cells together, supporting the barrier function (Awad et al., 2017). Consequences of leaky gut usually are local and systemic inflammation due to bacterial translocation to other organ sites (Rostagno, 2020; Dal Pont et al., 2020), impairment of nutrient absorption due to the damaged intestinal barrier, and ultimately, impacting feed conversion, growth rate, and productivity of the bird (Wideman, 2016; Awad et al., 2017; Cuccato et al., 2022; Santos et al., 2021; Ducatelle et al., 2023).

#### Dietary fiber and intestinal integrity

Dietary fiber plays a vital part of poultry diets, and its inclusion has significant positive impacts on intestinal integrity, digestion, and physiological health of the bird/flock (Table 3). Dietary fiber functions as a substrate for beneficial bacteria in the gut; thus, inducing a health-modulating fermentation process that generates short-chain fatty acids, lowers gut pH, suppresses pathogenic microbes, and contributes to improved intestinal integrity and overall host well-being.

**Table 3:** Effects of dietary fiber on intestinal integrity in poultry.

Positive	Negative
Stimulate growth/development of gut	Excessive/poor quality NSP^b^ = viscous environment – reduces nutrient absorption and digestion
Strengthen mucosal structure and function	Excessive/poor quality (NSP) = impairs bird growth – impaired nutrient uptake/utilization
Act as substrate for beneficial gut microbes = enhance immune response	Act as substrate for beneficial gut microbes. Excessive/poor quality (NSP) = inflammatory = **leaky gut**
Increase villus height = nutrient absorption	Excessive/poor quality can cause gizzard erosion
Increase production of SCFA^a^ by microbiota = improves gut health and increases enterocyte energy metabolism	

a Short chain fatty acids.

b Non-starch polysaccharides.

Further, DF stimulates growth/development in small and large intestines, improves intestinal structure and morphology by increasing villus height which enhances nutrient absorption, and increases fermentation by beneficial bacteria which produces SCFAs resulting in improved gut health and increased intestinal epithelial energy metabolism (Singh and Kim, 2021; Jha and Mishra, 2021). To ensure fiber helps rather than hinders digestion, it is necessary to consider the source, the inclusion level, and functional characteristics like how it moves through the gut and interacts with microbes (Table 3; Jha and Mishra, 2021).

#### Biomarkers of poultry intestinal integrity

Biomarkers of poultry intestinal integrity are quantifiable indicators that signify the functionality and health of the gastrointestinal tract in the bird. Biomarkers are used to assess the impact of factors such as diet, infection, stress, and alternatives to antibiotics on the gut homeostasis (health) of the bird. Table 4 provides a brief historical perspective of the variety and types of biomarkers in poultry that have been evaluated in the last decade. In general, biomarker analyses have included invasive and non-invasive measures of inflammation, intestinal permeability, oxidative stress, and indicators of nutrient absorption and microbiota balance (Table 5).

**Table 4:** Historical evaluation of biomarkers of poultry intestinal integrity.

Authors/Year	Model	Biomarkers	Reference
Ducatelle et al., 2018	Review	Invasive and non-invasive markers	Vet. Res. 49:43
Goosens et al., 2018	Necrotic enteritis and coccidiosis	Fecal – ovatransferrin	Vet. Res. 49:51
De Meyer et al., 2019	Antibiotics + wheat/rye diet + bacterial cocktail + coccidiosis	Colonic contents: higher abundance compared to control animals in colonic content: alpha-actinin-4, annexin A5, apolipoprotein A-1, fibronectin, hemoglobin subunit beta, myeloid protein 1, nucleophosmin, ovoinhibitor, transthyretin Ileal contents: lower abundance compared to controls: superoxide dismutase [Cu–Zn], Angiotensin-converting enzyme, mitochondrial aspartate aminotransferase, cathepsin D, Ig lambda chain C region, Ig lambda chain V-1 region, TTR and WD repeat-containing protein 1	Vet. Res. 50:46
Celi et al., 2019	Review	Invasive and non-invasive markers	An. Feed Sci. Technol. 250:9
Baxter et al., 2019	Corn and rye-based diet	Serum – citrulline, interferon-γ Cloacal swab – IgA	Front. Vet. Sci. 6:144
Barekatain et al., 2020	Corn- or rye-based diets	Excreta – intestinal alkaline phosphatase, lipopcalin-2, fibronectin	Plos One 15:e0237505
Dal Pont et al., 2021b	Rice-based diet (high NSP)	Serum – calprotectin Fecal – calprotectin, lipocalin-2, Hypoxia inducible factor-1α	Front. Immunol. 12:676628
Williams et al., 2022	Coccidiosis	Cecal content and excreta: microRNAs	Avian Pathol. 51:395-405
Kogut et al., 2024	Rice-based diet (high NSP)	Fecal, duodenal and cecal contents, duodenal and cecal tissue: norepinephrine	Poult. Sci. 103:104061
Mantzios et al., 2024	*Campylobacter jejuni* infection	Serum – IL10, cortisol; Fecal – ovatransferrin; Intestinal tissue – Occludin, toll-k receptor-4, mucin mRNA	Pathogens 13:356
Pires et al., 2025	Review	Non-invasive markers	Res. Vet. Sci. 190:105699

**Table 5:** Summary of biomarkers evaluated for gut health in poultry.

Gut health category	Biomarker	Invasive (I) Non-invasive (NI)	Functional description
Intestinal permeability =Intestinal barrier function	FITC-dextran passage (FITC-d)	I = blood/serum	Measure passage from gut to bloodstream
	Intestinal fatty acid binding protein (I-FABP)	I = blood and NI = feces	Protein found in gut
	Intestinal alkaline phosphatase (IAP)	NI = feces	Enzyme produced in gut
	Ovatransferrin	NI = feces	Iron-binding protein in gut Antimicrobial ctivity
	Tight junction proteins (Claudin, Zonulin)	I = blood NI = feces	Bind intestinal epithelial cells together
Inflammation	Lipocalin	I = blood NI = feces	Acute phase protein released by intestinal epithelial cells and heterophils
	Calprotectin (Avian MRP-126)	I = blood NI = feces	Protein released from heterophils
	Lactoferrin	I = blood NI = feces	Iron-binding protein released by heterophils
	Cytokines (IL1β, IL6, IL10, TNF-α, etc.)	I = blood NI = feces	Proteins produced are indicators of inflammation
	Succinate, citrate, Isocitrate	I = blood NI = feces	Metabolic indicators of inflammation
	Hypoxia inducible factor-α	NI = feces	Transcription factor that regulates metabolic switch from OxPhos to glycolysis (immunometabolic marker of inflammation)
Oxidative Stress	Thiobarbituric acid reactive substances (TBARS)	I = blood, tissues	Marker of lipid peroxidation by reactive oxygen species
	Total antioxidant capacity (TAC)	I = tissues	Measure of overall ability to neutralize oxygen free radicals
	Superoxide dismutase (SOD) Glutathine peroxidase (GPx)	I = blood, tissue	Enzymes to assess ability t neutralize free radicals
Microbiota signatures	Short chain fatty acids (SCFAs)	I = blood NI = feces	Analytical tool to evaluate fermentation of dietary fiber by microbiota
	Firmicutes:Bacteroidetes ration (F/B)	I = gut scrapings NI = feces	Indicator of gut health
	Lipopolysaccharides (LPS)	I = blood, tissue	Component of gram negative bacteria; presence indicative of bacterial translocation

One issue with the establishment of clearly defined biomarkers of intestinal integrity of poultry has been the limited availability of large, comprehensive, and well-managed databases is fundamental for advancing poultry health monitoring, developing effective diagnostic and predictive tools, and ultimately ensuring the efficiency and sustainability of the poultry industry (Kasab-Bachi et al., 2017). Clearly, one of the main problems with developing large databases is that poultry populations exhibit significant variation in factors influencing biomarker levels, including age, breed, sex, environmental conditions (e.g., temperature, housing), diet, and management practices. Large databases can account for this inherent variability. Moreover, large datasets are crucial for validating the sensitivity and specificity of identified biomarkers and the diagnostic tools used to measure them. Accurately identifying what constitutes a normal or healthy range for a gut biomarker is essential for distinguishing healthy birds from those experiencing health challenges. Large databases provide the scope of data needed to define ‘normal ranges’ across diverse poultry populations and production systems, allowing for a more nuanced understanding of biomarker values.

## Session 5: Fiber and performance: connecting the dots for optimal performance

### Carrie L. Walk

Dietary fiber was first described in 1953 in relation to pregnancy toxemia in women between 1940 and 1950 (Hipsley, 1953). Interestingly, today we are still finding relationships between DF and reproductive performance. For example, Dr Petry (section 2) describes the benefits of DF in sows to improve satiety and insulin sensitivity, reduce constipation and farrowing duration, and improving milk yield during lactation. Beneficial effects of DF on microbial fermentation, gizzard function, intestinal morphometry, immune function, satiety, and behavior in layers, breeders, or broilers have also been observed (Desbruslais et al., 2021; Enting et al., 2007; Kheravii et al., 2018; Zuidhof et al., 1995). However, the beneficial effects of DF are not unequivocal. Dietary fiber is also considered an anti-nutrient with impacts on digesta viscosity, entrapment of nutrients and reduced digestibility, and diluting the nutrient density of the diet. Some of this confusion is related to the complexity of DF and the lack of precision when using the term DF.

As analytical techniques improve, so does our knowledge of DF and the major components that are described within the term (i.e. total, soluble, and insoluble non-starch polysaccharides [**NSP**], and lignin) and their concentration within cereals, protein meals, and by-product meals used in animal feed. The use of accurate, reliable, and cost-effective NIRS to measure the NSP and lignin content in feed ingredients will enable real-time, accurate feed formulation, targeting ingredient concentrations to provide beneficial concentrations of these fractions in the diet, as well as improve enzyme efficacy and recommendations (Gomes et al., 2020; Nieto-Ortega et al., 2022). This means diets could be formulated to include feed ingredients or additives with specific NSP or oligosaccharide fractions to improve feed conversion ratio (**FCR**), promote beneficial bacterial fermentation, gut function through production of SCFA and minimize protein fermentation and pathogenic bacterial growth. For example, feeding increasing concentration of free xylose or free arabinose in broiler diets significantly increased FCR (Schutte, 1990) and increasing the concentration of soluble arabinoxylan from 0.4 to 1.2% in diets fed to young broilers linearly increased FCR (Gomes et al., 2021). However, in older birds, supplementation of increasing concentrations of soluble arabinoxylan from 0.4 to 1.2% linearly improved FCR. The previous results indicate feed formulation using NSP profiles targeted to optimize FCR or minimize detrimental impacts of NSP are possible with rapid and reliable methods to measure NSP content in ingredients and diets. Furthermore, supplementation of a stimibiotic to improve fiber degradation resulted in improvements in FCR in broilers at both ages, especially as the soluble arabinoxylan content increased in the diets (Gomes et al., 2021). Stimbiotics are a new class of non-digestible feed additives, described by Gonzalez-Ortiz et al. (2019) to promote dietary fiber utilization by the commensal microbial community. They are added to the diet at low doses (50–100 g/t) and therefore could not have direct impacts on fermentation but stimulate fiber-digesting bacteria to become more active and utilize fiber in the diet (Ribeiro et al., 2018). The previous findings give initial insights into the challenges and benefits of formulating diets to meet specific DF or more importantly NSP requirements and further improvements in efficiency that can be achieved with feed-additives to improve fiber degradation.

As well as measuring the benefits of the DF fractions on animal performance and microbial populations, the use of non-invasive biomarkers may provide an insight into the effects of DF fractions on intestinal integrity, nutrient utilization, and immune function and resilience, especially under enteric challenges such as coccidiosis, E coli, or *Clostridium perfringens*. Rinttila and Apajalahti (2013) observed relationships between cecal bacterial compositions and FCR on commercial farms in Northern Ireland and Finland. Similarly, Bernard et al. (2024) observed hens fed a higher fiber diet had a richer and more diverse microbiome and this was associated with improvements in hen feed efficiency.

When selecting biomarkers, numerous options and methods are available. The ideal markers should be obtained using non-invasive methods and rapid, reliable, and preferably cheap assays. The markers themselves should be specific, sensitive, and robust (Ducatelle et al., 2018). In most cases, more than one biomarker would be ideal to provide a holistic overview of nutritional, environmental, and host factors that may be contributing to the phenotypic response noted in the animal. For example, evaluation of a combination of the relevant commensal bacterial communities, SCFA produced by those communities, presence of pathogenic bacteria, and markers of inflammation and intestinal integrity may provide a holistic overview of the gastrointestinal environment. These results may provide insights into dietary or husbandry factors that can be amended to improve growth performance and feed efficiency. For example, coccidiosis challenge decreased microbial diversity in the ceca of broilers (Graham et al., 2023) and greater microbial diversity was previously associated with improved efficiency in layers (Bernard et al., 2024). Furthermore, piglets fed 6% wheat bran had a greater concentration of calprotectin (a marker of inflammation) compared with piglets fed 0 or 3% wheat bran or stimbiotic (Batista Kuneff et al., 2025). These results indicate comparison of biomarkers with healthy or good performing animals or flocks will provide greater insights into enteric challenges and effects of DF on gut function and immune responses. As our understanding and ability to measure DF and its fractions improves, so does our ability to harness the beneficial effects of DF, mitigate detrimental effects with optimal feed formulations, exogenous enzymes, and feed additive supplementation, and maintain intestinal homeostasis and function for optimal performance, as measured using biomarkers and linking that to optimum phenotypic responses.

## Conclusion

Dietary fiber is no longer a simple diluent of energy but a complex, functional nutrien**t** whose effects depend on source, chemical structure, physicochemical properties, and inclusion level. Advances in analytical techniques (e.g., TDF quantification, NIRS) and an improved understanding of fiber–microbiome–host interactions allow nutritionists to move beyond CF metrics toward precision DF utilization. Translational lessons from swine demonstrate that tailored DF strategies can enhance satiety, reproductive efficiency, welfare, and gut health in poultry, particularly in breeders and layers. However, the successful integration of DF into modern poultry feeding programs requires**:**

• Comprehensive fiber profiling, including soluble and insoluble fractions and their hydration properties.
• Strategic matching of DF type to production stage, disease pressure, and desired physiological outcomes.
• Advanced biomarkers to monitor intestinal integrity and microbiome shifts in real time.
• Data-driven models to predict DF responses across diverse genotypes and environments.

A system-based, cross-species approach, combining feed ingredient characterization, functional microbiome analysis, gut integrity biomarkers, and performance metrics will be critical to fully unlock DF’s potential in sustainable, welfare-conscious poultry production.

## CRediT authorship contribution statement

**Caitlin Evans:** Writing – original draft, Writing – review & editing. **Amy Petry:** Writing – original draft, Writing – review & editing. **Tim Johnson:** Writing – original draft, Writing – review & editing. **Michael H. Kogut:** Writing – original draft, Writing – review & editing. **Carrie L. Walk:** Conceptualization, Writing – original draft, Writing – review & editing. **Tara York:** Conceptualization, Writing – original draft, Writing – review & editing, Supervision, Project administration.

## Disclosures

All authors on the manuscript, “A New Focus On Fiber in poultry: Is it a nutrient or antinutrient” (Caitlin Evans, Amy Petry, Tim Johnson, Michael H. Kogut, Carrie L. Walk, Tara York) have no conflicts of interest to declare for this manuscript.
